# Diverse dynamics features of novel protein kinase C (PKC) isozymes determine the selectivity of a fluorinated balanol analogue for PKCε

**DOI:** 10.1186/s12859-018-2373-1

**Published:** 2019-02-04

**Authors:** Ari Hardianto, Varun Khanna, Fei Liu, Shoba Ranganathan

**Affiliations:** 1 Department of Molecular Sciences, Macquarie University, Sydney, NSW 2109 Australia; 2 Department of Chemistry, Universitas Padjadjaran, Jatinangor, West Java 45363 Indonesia; 3 School of Medicine, Faculty of Medicine, Nursing and Health Sciences, Flinders University, Adelaide, SA 5042 Australia

**Keywords:** Fluorinated balanol analogue selectivity, PKCε, Novel PKC isozymes, Unique dynamics feature, Molecular dynamics simulations

## Abstract

**Background:**

(−)-Balanol is an ATP-mimicking inhibitor that non-selectively targets protein kinase C (PKC) isozymes and cAMP-dependent protein kinase (PKA). While PKA constantly shows tumor promoting activities, PKC isozymes can ambiguously be tumor promoters or suppressors. In particular, PKCε is frequently implicated in tumorigenesis and a potential target for anticancer drugs. We recently reported that the C5(*S*)-fluorinated balanol analogue (balanoid **1c**) had improved binding affinity and selectivity for PKCε but not to the other novel PKC isozymes, which share a highly similar ATP site. The underlying basis for this fluorine-based selectivity is not entirely comprehended and needs to be investigated further for the development of ATP mimic inhibitors specific for PKCε.

**Results:**

Using molecular dynamics (MD) simulations assisted by homology modelling and sequence analysis, we have studied the fluorine-based selectivity in the highly similar ATP sites of novel PKC (nPKC) isozymes. The study suggests that every nPKC isozyme has different dynamics behaviour in both apo and **1c**-bound forms. Interestingly, the apo form of PKCε, where **1c** binds strongly, shows the highest degree of flexibility which dramatically decreases after binding **1c**.

**Conclusions:**

For the first time to the best of our knowledge, we found that the origin of **1c** selectivity for PKCε comes from the unique dynamics feature of each PKC isozyme. Fluorine conformational control in **1c** can synergize with and lock down the dynamics of PKCε, which optimize binding interactions with the ATP site residues of the enzyme, particularly the invariant Lys437. This finding has implications for further rational design of balanol-based PKCε inhibitors for cancer drug development.

**Electronic supplementary material:**

The online version of this article (10.1186/s12859-018-2373-1) contains supplementary material, which is available to authorized users.

## Background

**Fig. 1:**
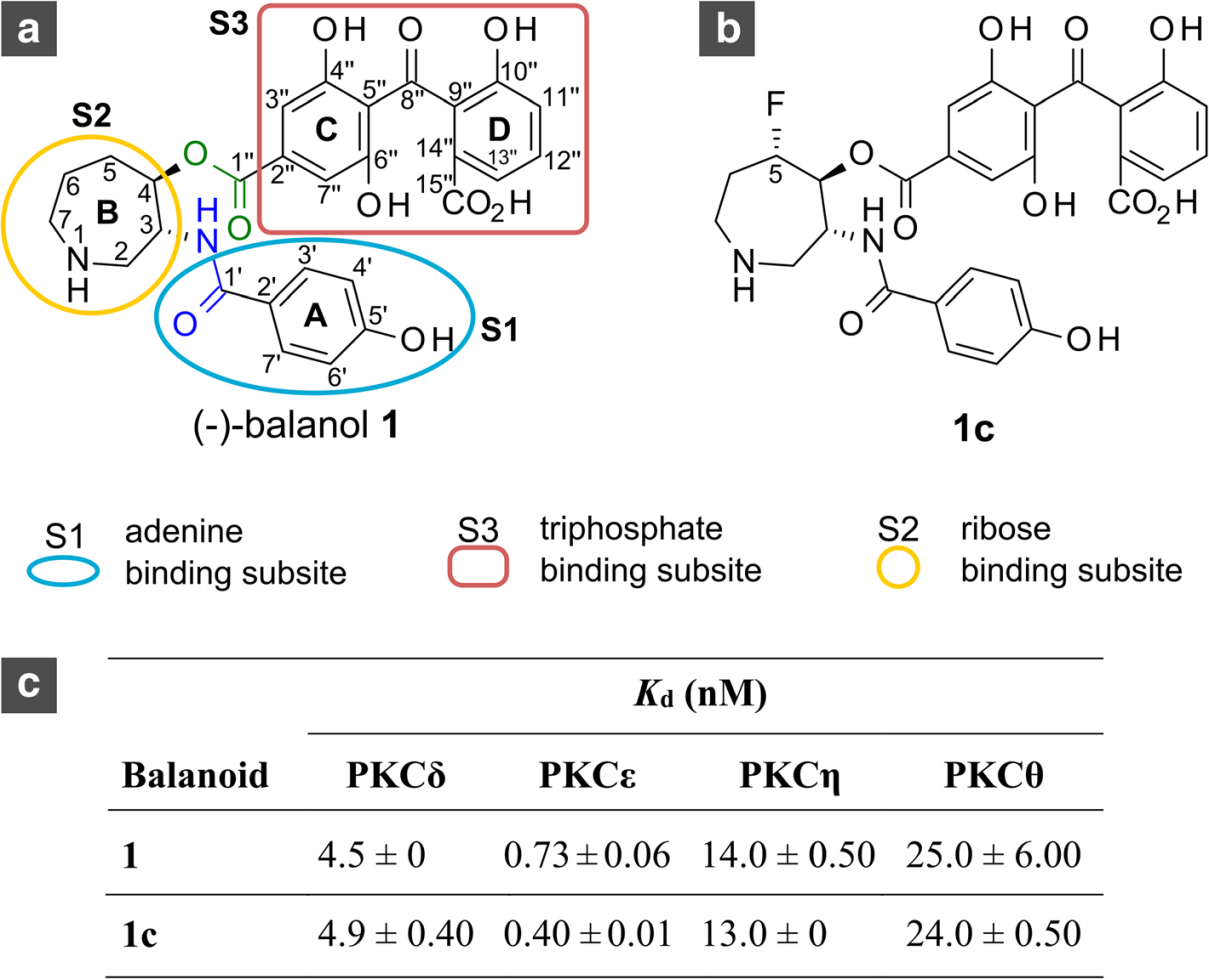
Structures of **a** (−)-balanol and **b** its C5(S)-fluorinated analogue (**1c**) with **c** experimental binding affinity to novel PKC isozymes. Each structure consists of three moieties which reside three different subsites based on structural superimposition of balanol and ATP

(−)-Balanol (referred to as balanol) is an ATP-mimicking inhibitor [1] from a fungus *Verticillium balanoids* [2]. Its structure consists of three moieties, the benzamide (ring A), azepane (ring B), and benzophenone moieties (ring C and D). These moieties completely fill the flexible ATP site (Fig. 1a) [2]. The benzamide and azepane moieties occupy the adenine and ribose subsites, respectively, whereas the benzophenone moiety resides in the triphosphate subsites. In balanol structure, the azepane ring is in a central position [3] that connects the benzamide and benzophenone moieties. Amide and ester linkages respectively join the benzamide and benzophenone moieties to the azepane.

Balanol is a non-selective inhibitor for cAMP-dependent protein kinase A (PKA) and protein kinase C (PKC) isozymes that target their ATP sites [4]. While PKA is known to promote tumor [5], most PKC isozymes show ambiguous roles in cancer, i.e. they can act as tumor promoters or suppressors, depending on the context [6]. For instance, PKCβI suppresses breast cancer, whereas PKCα, PKCβII, and PKCδ promote the cancer [6]. In contrast, PKCβI and PKCδ respectively are promoter and suppressor in prostate cancer. In particular, PKCε is consistent oncogene protein and potentially targeted for anti-cancer drugs [6]. Thus, improving balanol selectivity to PKCε is essential for development of anti-cancer inhibitor. However, development of isozyme-specific inhibitors are elusive due to the highly homologous ATP sites of PKC isozymes, and protein kinases in general [7].

Extensive structure and activity relationship (SAR) studies [8–12] have been carried on balanol to improve its selectivity to certain PKC isozyme, with PKA used as a reference in some studies [9, 10, 12]. These SAR studies investigated chemical modifications on the benzamide [8], azepane [9], and benzophenone moieties [10–12] (Fig. 1a). Modifications on the benzamide moiety showed that the phenolic C5′OH group is critical for PKC inhibition [8], whereas derivatizations on the benzophenone moiety revealed the importance of the acidic functional group [10, 11]. In some SAR studies [8, 9], a five-membered pyrrolidine ring replaced the azepane ring but did not substantially improve the selectivity of balanol. The studies showed that the azepane ring can compromise various derivatization, but selectivity is still a challenge [10].

Fluorine is a unique element which has been widely employed in drug development [13, 14]. It can substitute hydrogen in an organic molecule without considerably increasing molecular size due to its small atomic size, 1.47 Å which is closer to 1.20 Å for hydrogen. Its highly electronegative character (Pauling electronegativity of 4.00) causes fluorine forming an extremely polarized C–F bond. Such bond has a significant ionic character and creates a low-energy σ* antibonding orbital which can receive electron density from lone pairs or σ-bonds. These unique characters make the C–F bond conveying conformational effect through dipole–dipole interactions, charge–dipole interactions, and hyperconjugation that can drive molecular shape [15]. Appropriate fluorination can yield fruitful effects on conformation, intrinsic potency, p*K*_a_, metabolic pathways, membrane permeability, and pharmacokinetic properties [13]. Hence, fluorination may bestow a protein kinase selectivity to balanol.

In a recent study [3], for the first time, we carried out stereospecific single and multiple fluorinations on the azepane moiety of balanol. The effect of azepane fluorination on protein selectivity was evaluated by binding affinity measurement of balanol and the fluorinated balanol analogues (altogether referred as balanoids) to novel PKC (nPKC) isozymes and PKA [3]. We found that the fluorinations on balanol produce various responses to kinases studied, where the C5(*S*)-fluorinated analogue or **1c** (Fig. 1b) exhibits an improved binding affinity and selectivity to PKCε (see Fig. 1c) over other nPKC isozymes and PKA. This stereospecific fluorination offers ligand-enzyme selectivity in highly homologous ATP sites [3].

Understanding the key determinant behind the selectivity of **1c** for PKCε will be beneficial for future development of ATP mimic inhibitors based on balanol. This understanding can be achieved rapidly using computational approaches, such as molecular dynamics (MD) simulations. MD simulations offer thorough analysis of the interaction of **1c** with the ATP site residues [16] to uncover selectivity of **1c** to PKCε. MD also allow to study the conformational change of ligand and estimate the binding energy from ensemble conformations. Furthermore, this method gives an opportunity to investigate induced-fit interactions in ligand binding [17], since protein kinases possess such plasticity [1]. In our previous work by exploiting the power of MD [18], we revealed an interesting factor causing different binding responses of PKA and PKCε to balanoids. Those different binding responses are contributed by a structurally equivalent residue in each kinase, Thr184 in PKA and Ala549 in PKCε.

Here, we present a MD study assisted by homology modelling and sequence analysis of **1c** in nPKC isozymes to unravel selectivity of the balanoid to PKCε. For the first time to the best of our knowledge, we found that the unique dynamics feature of each nPKC isozyme lead to **1c** selectivity to PKCε. Although the ATP site residues among nPKC isozymes are highly similar, the other residues are relatively low conserved which differently organize residues in the ATP site and shape the pocket. As a result, the dynamics responses of nPKC isozymes to **1c** are diverged and yields various binding affinity to the balanoid. Furthermore, fluorine conformational control in **1c** exhibits better synergy with PKCε than the other nPKC isozymes, resulting in an optimized interaction and a selectivity. Finally, this study offers beneficial information for the future rational design of balanol analogues to achieve an improved binding affinity and isozyme selectivity to PKCε.

## Methods

### Homology modelling

Using a homology modelling approach as described previously, we built kinase domains of human nPKC isozymes which bind balanol (for Uniprot ID [19], see Additional file 1: Table S1). In modelling process, we used either single or multiple templates as listed in Table S1 (Additional file 1). To examine the structural conservation between both templates, we carried out structural alignment using MultiSeq [20] as implemented in Visual Molecular Dynamics (VMD) 1.9.2 [21]. In the next step, we utilized CLUSTALX 2.1 [22] to align the query to template sequences. Moreover, we manually edited the resulting sequence alignment to map the ‘open’ conformation [23] of the Gly-rich loop (GXGXXG) from 1BX6 to PKCε model. Percentage of sequence similarity and identity of kinase domains and the ATP sites were calculated using MatGAT [24].

MODELLER 9.14 [25] was employed to perform homology modelling. The modelling stage included assigning balanol from mouse PKA-balanol (PDB ID: 1BX6) to the generated query structure. This step is to retain the structural features of the ATP binding site in the resulting model. Subsequently, Discrete Optimized Protein Energy (DOPE) score [26], which reflects the quality of the model, was then used to evaluate the resulting models. Furthermore, we structurally aligned models from multiple runs of homology modelling and chosen the structure with residues adopting consensus conformations. The selected model was also assessed using Ramachandran plot as implemented in PROCHECK [27].

### Clustering of ATP site interfaces

We utilized the webPIPSA [28] server to cluster ATP site interfaces of nPKC isozymes based on molecular surface electrostatic potential (MSEP). Initially, the web-server computed the MSEPs of the ATP site interfaces calculation using the University of Houston Brownian Dynamics (UHBD) program [29]. On the second step, webPIPSA used the PIPSA algorithm to compare MSEPs and calculate electrostatic distances. Subsequently, the electrostatic distances were clustered and portrayed as a heat map using the R statistical software [30].

### Molecular dynamics simulation preparation and protocol

Since fully activated human PKC isozymes are phosphorylated at specific sites [19], we added phosphate groups on the kinases studied here at particular sites (Additional file 1: Table S1) using Discovery Studio Visualizer [31]. The initial ligand conformations were adopted from the natural balanol in 1BX6 (mouse PKA with bound balanol). Atomic charges of balanoids were determined using the Austin Model 1 - Bond Charge Corrections (AM1-BCC) [32] method in AmberTools16 [33]. Parameters for balanoids were derived from the General Amber Force-fields (GAFF) [34] and determined using the *parmchk* program in AmberTools16. We used ff14SB [35] to assign force-fields for regular amino acid residues and their side chains, whereas phosaa10 [36] was utilized for phosphorylated residues.

The preparation step of MD system was performed using the *tleap* utility in AmberTools16. We solvated each complex of each kinase-balanoid complex with explicit water molecules (TIP3P), where minimum distance between the protein and box boundary was 10 Å. To achieve neutral charge and a salt concentration of 0.15 M, which is the equivalent of physiological salt concentration, we added sufficient Na^+^ and Cl^−^ ions to the system.

GPU-accelerated Particle-Mesh Ewald Molecular Dynamics (PMEMD), as implemented in Amber16 [33], was employed throughout simulations where periodic boundary conditions were applied. For each simulation, two consecutive steps of energy minimization were carried out with restraining protein-ligand complex by 25 and 5 kcal.mol^− 1^.Å^− 2^. For 50 ps, the system temperature was then elevated to 300 K under NVT condition and then followed by equilibration steps. In the next 50 ps NPT simulation, the system density was equilibrated to 1 g.cm^− 1^. In the subsequent NVT simulation, the restraint on protein-ligand complex was gradually removed every 50 ps by 1 kcal.mol^− 1^.Å^− 2^ from 5 kcal.mol^− 1^.Å^− 2^ where in the last equilibrium step of 50 ps, the restraint was eliminated.

Production-phase was simulated under the NPT at 300 K. Particle-mesh Ewald (PME) [37] method was employed to handle long-range electrostatic interactions. We set 10 Å cut-off for short-range non-bonded interactions which was also employed by others [38, 39]. To constrain all bonds involving hydrogen atoms, we implemented a SHAKE algorithm [40]. Meanwhile, we implemented algorithms of Berendsen barostat [41] and Langevin thermostat [42] to maintain constant pressure and temperature, respectively. Convergence of simulations were checked by monitoring cosine contents of the first few principal components using GROMACS [43].

### Binding energy calculation

We calculated experimental binding energy values ($\Delta  {G}_{exp}^{{}^{\circ}}$) of balanoid **1c** to nPKC isozymes and PKA from dissociation constant (*K*_d_) values (Fig. 1c) [3]. At equilibrium and under standard conditions, the binding energy directly relates to the equilibrium constants and, thus, can be determined using the following equation (eq. 1):

1 $$ \Delta  {G}_{exp}^{{}^{\circ}}=- RTln\left({K}_a\right)= RTln\left({K}_d\right) $$

where $\Delta  {G}_{exp}^{{}^{\circ}}$ denotes experimental binding energy, respectively *K*_a_ and *K*_d_ are association and dissociation constant, R is the universal gas constant, and T is absolute temperature.

Molecular Mechanics Generalized Born Surface Area (MMGBSA) method [44], which is implemented in MMPBSA.py [45], was utilized to determine estimated binding energy values of balanoid **1c** to nPKC isozymes or PKA as well as their per-residue decomposition (Additional file 1: Note 2). MMGBSA does not incorporate conformational entropy or the free energy of water molecules in the binding site, although these components may have a role in protein-ligand interactions. Nevertheless, the MMGBSA method has applied successfully to rationale experimental data and to improve the results of virtual screening and docking [46]. The MMGBSA binding free energy ($\Delta  {G}_{MMGBSA}^{{}^{\circ}}$) is calculated as follows in eq. 2:

2 $$ \Delta  {G}_{MMGBSA}^{{}^{\circ}}={\left\langle {G}_{com}\right\rangle}_i-{\left\langle {G}_{rec}\right\rangle}_i-{\left\langle {G}_{lig}\right\rangle}_i $$

where 〈*G*_*com*_〉_*i*_, 〈*G*_*rec*_〉_*i*_, and 〈*G*_*lig*_〉_*i*_ are the average value of $\Delta  {G}_{MMGBSA}^{{}^{\circ}}$ for complex, enzyme, and ligand, respectively, over snapshots *i* extracted from MD trajectories. *G*_*x*_ can be decomposed as shown in the following eq. 3:

3 $$ {G}_x={E}_{MM}+{G}_{solv}^{GB}+{G}_{solv}^{SA} $$

where *E*_*MM*_ is the gas phase energy, ${G}_{solv}^{GB}$ is the electrostatic portion of solvation energy computed using Generalized Born (GB) implicit solvent model, and ${G}_{solv}^{SA}$ is the hydrophobic contribution to the solvation energy. The hydrophobic contribution was estimated using the Linear Combination of Pairwise Overlaps (LCPO) [47] method, whereas *E*_*MM*_ was approximated by the molecular mechanics energy of the molecule. Molecular mechanics energy consists of bond (*E*_*bond*_), angle (*E*_*angle*_), torsion energies (*E*_*torsion*_), van der Waals (*E*_*vdW*_), and electrostatic interactions (*E*_*el*_) (eq. 4).

4 $$ {E}_{MM}={\Sigma}_{bond s}{E}_{bond}+{\Sigma}_{angle s}{E}_{angle}+{\Sigma}_{torsion s}{E}_{torsion}+{\Sigma}_{i\ne j}^{atoms}{E}_{vdW}+{\Sigma}_{i\ne j}^{atoms}{E}_{electrostatic} $$

Internal energy terms, which include *E*_*bond*_, *E*_*angle*_, and *E*_*torsion*_, were omitted in this study, since the calculation was applied on single-trajectory MD simulations [48].

### Charge state validation

We adapted the charge state validation workflow as described previously [49]. Briefly, initial charge state of **1c** in each ATP site of nPKC isozyme refers to our previous work [49], that the amine (N1) on the azepane ring, the phenolic group (C6′′OH), and the carboxylate (C15′′O_2_H) on the benzophenone moiety bear charges. $\Delta  {G}_{MMGBSA}^{{}^{\circ}}$ value was computed from each trajectory of nPKC-bound **1c**. Here, we also used PKA-bound **1c** trajectory from our previous study [50] for an additional data point. The resulting $\Delta  {G}_{MMGBSA}^{{}^{\circ}}$ values were compared with experiment. A detailed description is presented in (Additional file 1: Note 1, Figures S1 and S2).

### MD trajectory analysis

Analyses were performed on trajectories after convergence was reached (Additional file 1: Table S2). To analyze MD trajectories, we utilized *cpptraj* program in AmberTools16 [33]. The analysis consisted of computation of dihedral angles in the azepane ring, H-bond conservation, Solvent-Accessible Surface Area (SASA), and Root-Mean-Square Fluctuation (RMSF). Principal component analysis (PCA) dynamics modelling was performed using ProDy [51] plugin in VMD 1.9.2 [21].

### Image and graph generation

Conformational ensembles of balanoid **1c** in the ATP sites of PKC isozymes were visualized in VMD 1.9.2 [21]. The conformations were generated every 1250 frames from the trajectories of the last 100 ns (5000 frames) for analysis purposes. Non-covalent interactions of kinase-balanoid were depicted using BIOVIA Discovery Studio Visualizer 2016 [31] by referring to H-bond conservation analysis. Two dimensional (2D) structures of balanoids were sketched in BIOVIA Draw 2016 [52]. All graphs were produced in RStudio 0.99.892 [53], utilizing the R statistical software package [30], ggplot2 [54]. For image editing, GNU Image Manipulation Program (GIMP) 2.8.14 [55] and Inkscape 0.48.5 [56] were used.

## Results and discussion

### General conformational ensemble of 1c in the ATP sites of novel PKC isozymes

**Fig. 2:**
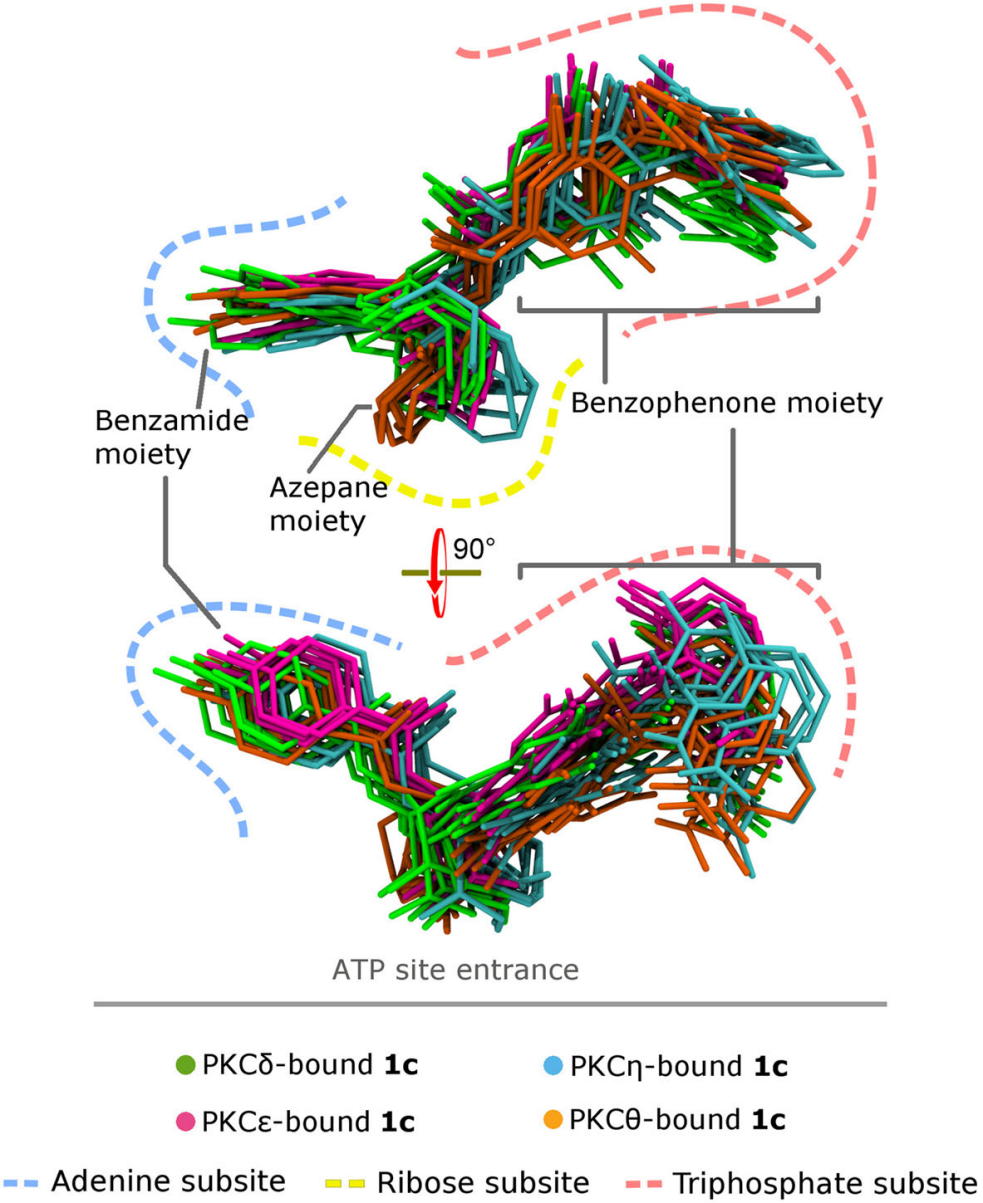
Superimposition of conformational ensemble of **1c** that is bound to nPKC isozymes (PKCδ, PKCε, PKCη, and PKCθ). On the upper panel, direction toward reader represents the direction to the entrance of the ATP site

According to superimposition of the conformational ensemble of **1c** that is bound to nPKC isozymes (Fig. 2), each moiety of **1c** exhibits docking variability in subsites of the ATP site, where the order is: the benzamide moiety < the azepane ring < the benzophenone moiety. While the benzamide part of **1c** still shows similarity, the azepane ring docks differently, in particular, the azepane rings of PKCη- (orange) and PKCθ-bound **1c** (cyan), which have *K*_d_ above 7 nM (Fig. 1.C). Interestingly, the azepane rings of PKCδ- (green) and PKCε-bound **1c** (magenta) with *K*_d_ below 5 nM (Fig. 1.C) are in a similar docking position. Additionally, the azepane ring of **1c** that is bound to PKCδ and PKCε has similar limited flexibility in such kinases, whereas the azepane ring is more fluctuated in PKCη and PKCθ (Fig. 2).

The benzophenone moiety of **1c** exhibits the most position diversity in the triphosphate subsites (Fig. 2). In PKCε, **1c** places its benzophenone moiety deeper inside the triphosphate subsite than that in nPKC isozymes, whereas PKCδ-bound **1c** shows its ring D swaying toward the subsite floor. In both PKCη and PKCθ, **1c** orients its ring D close to the entrance of the ATP sites where the benzophenone moiety has more flexible conformations in PKCη than PKCθ.

### Analysis of sequence alignment and clustering based on molecular surface electrostatic potential

The various ways of **1c** orienting its moieties may be due to unique environments of the ATP sites of nPKC isozymes. Therefore, we started to find the unique feature with sequence analysis (Additional file 1: Figure S3.A). We found that the ATP site residues, which directly contact with balanol, share a high sequence identity (93–96%) and similarity (100%) (Additional file 1: Table S3). Only two residues are different which are part of the adenine subsite (indicated by a green bar, Additional file 1: Figure S3.A), but they still retain similarities. We also conducted a cluster analysis on the ATP sites using molecular surface electrostatic potential (MSEP)-based methodology (webPIPSA server) [28]. The result also suggests that nPKC isozymes have very similar MSEP (Additional file 1: Figure S3.B).

Despite sharing high identity in their ATP sites, the whole kinase domain residues of nPKC isozymes are relatively less conserved with 59–76% identity and 77–90% similarity (Additional file 1: Table S4). As shown in the sequence alignment (the non-highlighted residues, Additional file 1: Figure S3.A), non-contact residues that support the ATP site are varied. These sequence variation among nPKC isozymes may be implicated in **1c** selectivity for PKCε.

### Dynamics feature of novel PKC isozymes

As noted above, although those kinases possess highly identical residues at the ATP binding site, sequence variation is observed on non-contact residues (Additional file 1: Figure S3.A). These varied residues may define structural dynamic features of nPKC isozymes and lead to unique responses of the kinases to **1c** binding. To evaluate this notion, we carried out separated MD simulations for *apo* forms of nPKC isozymes here and also compared them to their related **1c**-bound forms (hereafter refers as bound).

**Fig. 3:**
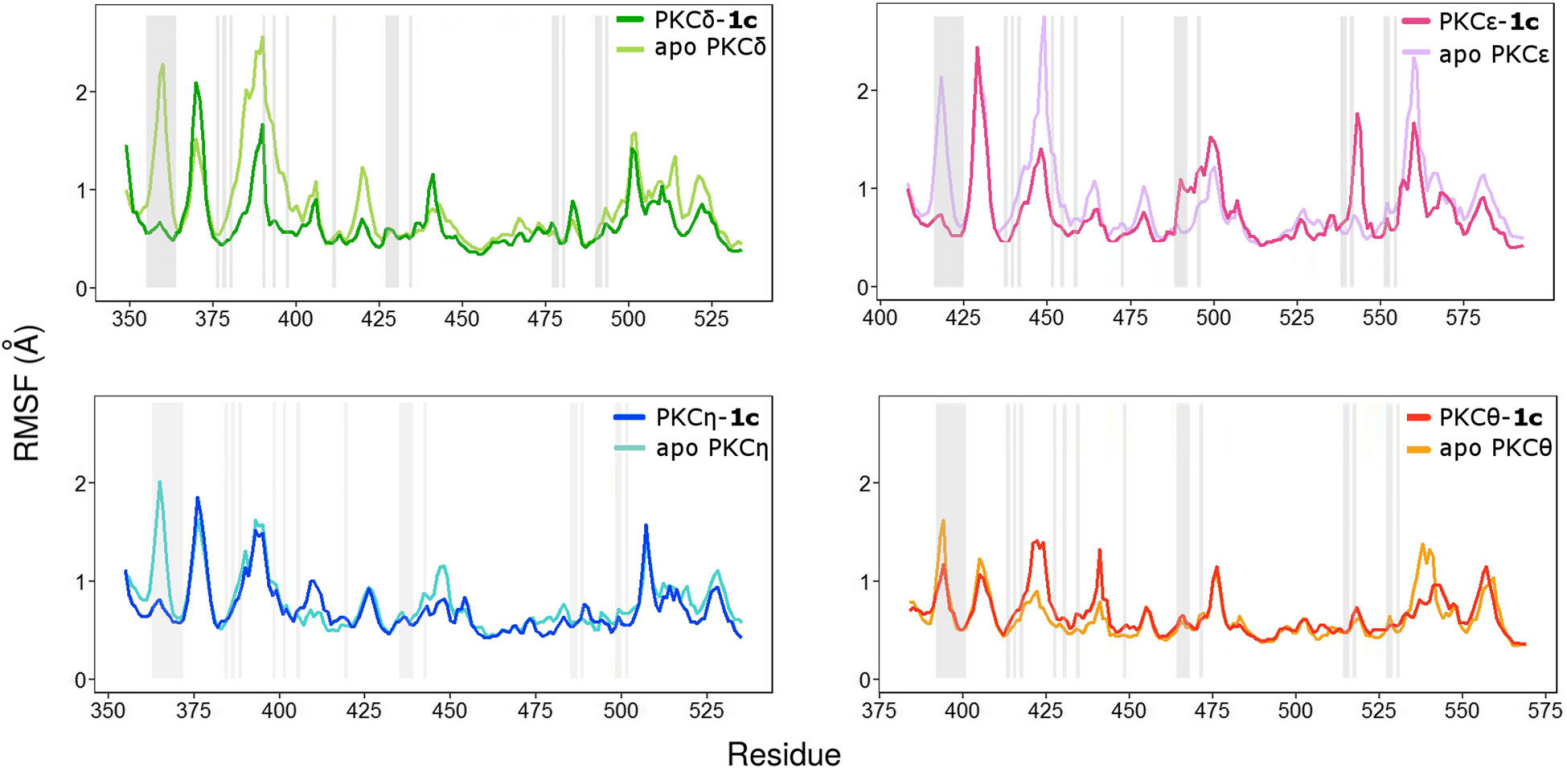
Root Mean Square Fluctuation (RMSF) plots of novel PKC isozymes in the *apo* and **1c**-bound forms. Root Mean Square Fluctuation (RMSF) plots of novel PKC isozymes in the *apo* and **1c**-bound forms. Residues of ATP binding sites are indicated by grey-shaded bars

As shown in RMSF plots (Fig. 3 and Additional file 1: Figure S4) and structural mapping of PCA dynamics modelling (Additional file 1: Figure S5), nPKC isozymes display different dynamics behavior. In their *apo* forms, they show diverse flexibility degree in both contact and non-contact residues of the ATP binding sites (Additional file 1: Figure S4.A). These results indicate that the variability on the non-contact residues among nPKC isozymes determine the dynamics of the contact residues, which may influence the responses and the interactions of the kinases to **1c**. RMSF plots of nPKC isozymes in bound forms (Additional file 1: Figure S4.A) and structural mapping of PCA dynamics modelling (Additional file 1: Figure S5) reveal dynamics alterations of the kinases after **1c** binding. Similar to the bound *apo* forms, the structural dynamics of the bound forms differ among nPKC isozymes, pointing out that each kinase responds differently to **1c** binding.

We subsequently performed a Wilcoxon rank sum test to evaluate RMSF differences between the *apo* and bound forms of the given kinases. The results suggest that all nPKC isoforms, except PKCθ, significantly reduce their conformational freedom after binding **1c** (Additional file 1: S5). Interestingly, PKCε, which shows a higher binding affinity to **1c** than the other nPKC isoforms (Fig. 1), has the most significant difference of conformational freedom between its *apo* and bound form on the ATP site (*p*-value = 6.27 × 10^− 3^; Additional file 1: Table S5). The **1c** binding to PKCε reduces the conformational freedom of the kinase domain by 0.31 Å on average (Additional file 1: Table S6).

**Fig. 4:**
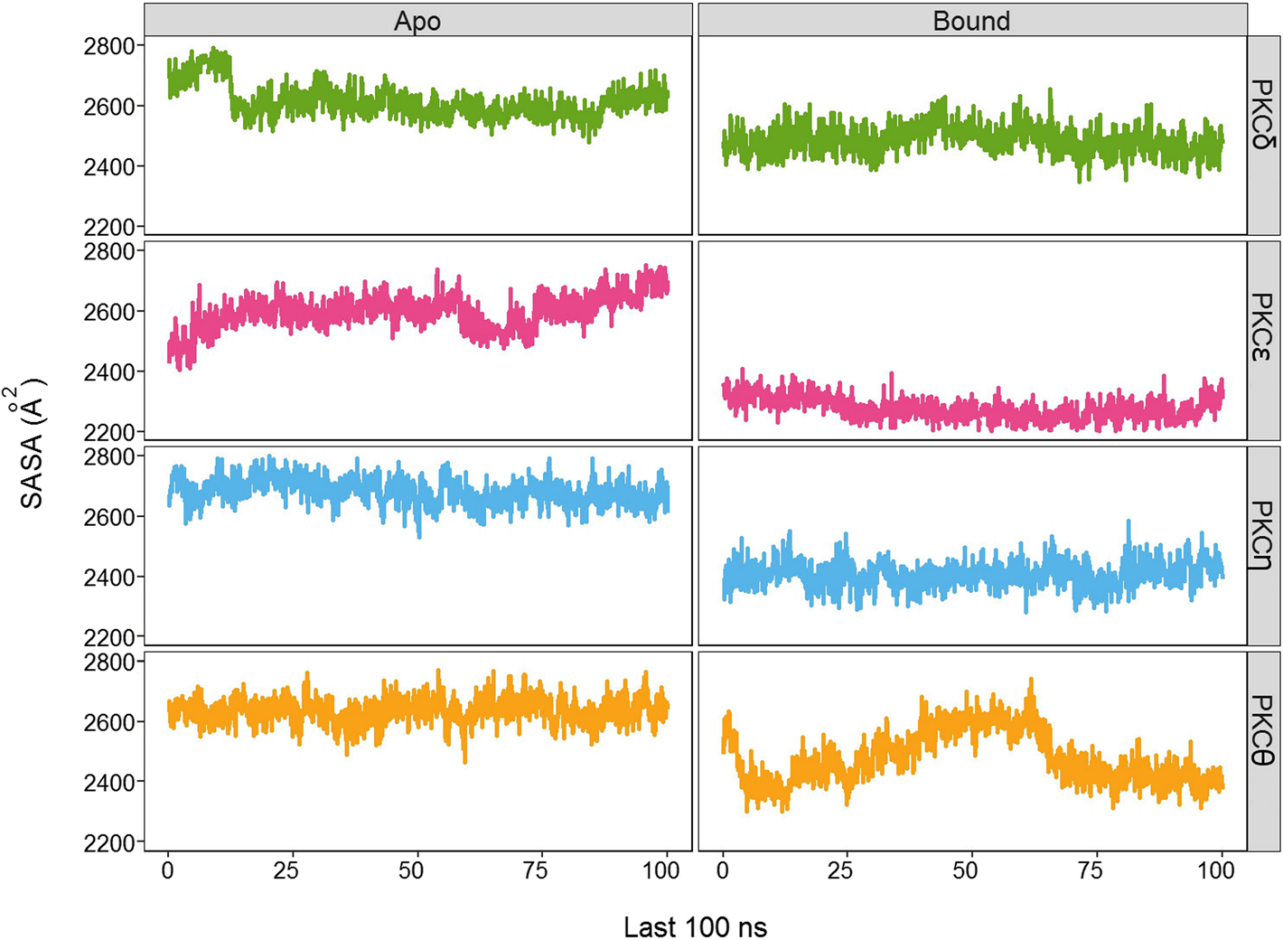
SASA plots. The plots show SASAs of both the *apo* and bound forms of nPKC isoforms from the last 100 ns of trajectories

We also conducted analysis of solvent-accessible surface area (SASA) at the ATP sites of nPKC isoforms in the *apo* and bound forms. All nPKC isoforms have slightly different SASA in their *apo* forms in terms of value and fluctuation (Fig. 4; Additional file 1: Table S7). Among nPKC isoforms, PKCε exhibits the most fluctuating SASA (Fig. 4), which is also reflected by its standard deviation (2598.62 ± 58.72; Additional file 1: Table S7). These data suggest that the variation on non-contact residues of the ATP sites among nPKC isoforms affects dynamics features and shapes the sites differently. Ultimately, these ATP sites diversely respond the **1c** binding, as shown in SASA values of nPKC isoforms in bound forms at the ATP sites (Fig. 4; Additional file 1: Table S7). One interesting finding here is that the bound form of PKCε, where **1c** is a strong binder, has the lowest and the least fluctuating SASA values among other nPKC isoforms (2276.31 ± 38.26 Å^2^; Additional file 1: Table S7).

These results support our hypothesis that the diversity on non-contact residues of the ATP sites may engender a unique dynamics feature for every nPKC isozyme. This uniqueness may differently organize the highly identical residues in the ATP site. As a result, the ATP site of individual nPKC isozyme may interact differently with **1c**.

### Interactions of the azepane ring at the ribose subsites

As discussed above, every nPKC isozyme possesses a unique dynamics feature that may diversely positions residues of the ATP site. As a consequence, those residues will interact differently with **1c** and explain the various conformational ensembles of **1c** in the ATP sites of nPKC isozymes (Fig. 2). Therefore, we elucidate interactions made by **1c** in every nPKC isozyme, particularly interactions between the azepane ring and the ribose subsite. This selection is based on an assumption that the azepane ring is the central moiety that connects the benzamide and the benzophenone moieties [3]. Furthermore, the azepane ring is the moiety where the fluorine substituent is incorporated at the C5(*S*) position. Any conformational changes on the azepane ring may interfere the other moieties as monitored in polar charts of the dihedral angle ω_6_ (Additional file 1: Figure S6). This interference may further influence interactions between the whole **1c** molecule and the ATP site.

**Fig. 5:**
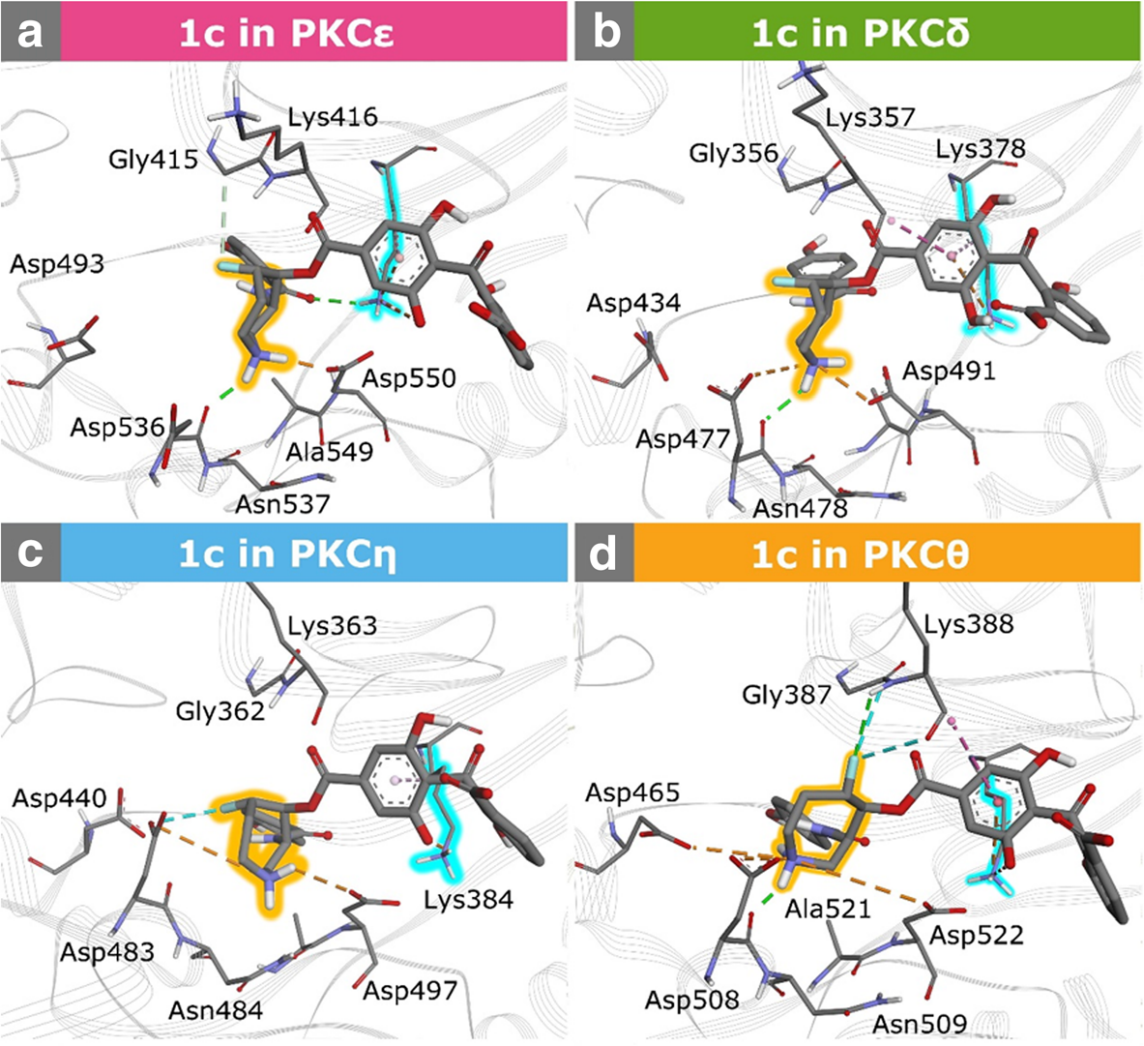
Interactions of the azepane rings of **1c** with the ribose subsite residues of novel PKC isozymes. **a** and **b** respectively depict interactions of **1c** in PKCε and PKCδ, whereas **c** and **d** show interactions of 1c in PKCη and PKCθ, respectively. The invariant Lys and the azepane ring are highlighted in cyan and orange, respectively. H-bonds are represented by green dashed lines, whereas salt bridges as well as π-cation interactions are depicted by orange dashed lines. Each conformation is a representative snapshot from the respective simulation

We start with **1c** in PKCε which exhibits the highest binding. The azepane ring of **1c** builds H-bonds with Asp536 and Asp550 via its N1 amine group (Fig. 5a), with conservations of 30.8 and 66.0% (Additional file 1: Table S8), respectively. These interactions help to shape and rigidify the conformation of the azepane ring (Fig. 2). As a result, the azepane ring less interferes the other moieties as seen in the trajectory of dihedral angle ω_6_ (Additional file 1: Figure S6). Furthermore, 1c can strengthen its interaction with the invariant Lys437 (Fig. 5a; Additional file 1: Note 2), which is considered as the most contributing residue in the binding of balanoid to kinases [18]. Balanoid 1c creates a very strong binding energy with the Lys437 (− 16.83 kcal.mol^− 1^; Additional file 1: Figure S7). The synergy between **1c** and the dynamics feature of PKCε results in interactions that are free from unfavorable binding energy (Supporting Information: Fig. S7), explaining the strong binding affinity of **1c** to the kinase [18].

In PKCδ, Asp536 and Asp550 correspond to Asp477 and Asp491. Interactions of N1 with Asp477 and Asp491 provide H-bond conservation of 46.4 and 99.3% each (Additional file 1: Table S8). These interactions stabilize the binding of **1c** to the ATP site of PKCδ by − 18.01 and − 6.06 kcal.mol^− 1^ for Asp491 and Asp477, respectively. Nonetheless, these stabilizations are violated by an unfavourable binding with Lys475 (0.19 kcal.mol^− 1^; Additional file 1: Figure S7). In addition, the uncharged phenolic C6′′OH group (Additional file 1: Note 1) causes less options to interact with the invariant Lys378. As a consequence, the Lys378 only provides a binding contribution to **1c** by − 8.79 kcal.mol^− 1^ (Additional file 1: Figure S7). Furthermore, the benzamide moiety of **1c** obtains weaker binding contributions in PKCδ than other nPKC isoforms (Additional file 1: Figure S7).

**1c** in PKCε, PKCη, and PKCθ has a same ionization state. Its azepane ring, however, exhibits different conformations (dihedral angles ω_1_-ω_6_; Additional file 1: Figure S6) and interaction modes with the ribose sites (Fig. 5). In PKCη, the azepane ring forms attractive charge and carbonyl-fluorine interactions with Asp483 (Fig. 5). Additionally, it builds a low conserved H-bond with Asp497 through the N1 amine group (Additional file 1: Table S8). Although these interactions can help to limit flexibility of the azepane ring in the ribose subsite, **1c** acquires relatively a weaker binding contribution from the invariant Lys384 (− 6.34 kcal.mol^− 1^; Additional file 1: Figure S7). Moreover, a repulsive interaction emerges from Glu403 (0.14 kcal.mol^− 1^) which weakens the binding of **1c** to PKCη.

For the case of **1c** binding to PKCθ, residues at the ribose subsite allow the azepane ring to have conformational flexibility (Fig. 2; Additional file 1: Figure S6). The N1 amine group in the azepane ring and Asp508 forms a H-bond with a conservation of 41.9% (Additional file 1: Table S8). Nevertheless, the amine group is also involved in attractive interactions with the side chains of Asp465, Asp508, and Asp522 (Fig. 5) which may contribute to the flexibility of the azepane ring. Moreover, the fluorine atom interacts with the backbones of Gly387 and Lys388 (Fig. 5). Such situation may contribute to the weak interactions of the benzophenone moiety with the triphosphate residues. For example, the benzophenone has a relatively weak binding with the invariant Lys409 and repulsive interactions with Asp421 and Glu428 (Additional file 1: Figure S7).

The interaction analysis above suggests that the unique dynamics feature of each novel PKC isozyme differently organizes its highly identical ATP site residues. As a result, **1c** interacts with every nPKC in different modes. While interactions of **1c** to PKCδ, PKCη, and PKCθ generate repulsive interactions that weaken the binding affinities to those kinases, this balanoid and PKCε interact in harmony (Additional file 1: Figure S8). The binding of **1c** to PKCε can optimize interactions to yield a strong affinity, in particular with invariant Lys437. Additionally, the unique behavior PKCε provides interactions with **1c** that are free from unfavorable binding energy.

## Conclusions

Here, we uncover different binding affinities of a C5(*S*)-fluorinated balanol analogue (**1c**) to the highly identical ATP sites of novel PKC isozymes. Such disperse binding affinities are due to the unique dynamics feature of every isozyme. This unique dynamics feature comes from the less identical residues beyond the ATP site which differently organizes the highly identical ATP site residues. As a result, residues in each ATP site of novel PKC isozyme interact differently with **1c**. The balanoid **1c**, that carries fluorine perturbation at the C5(*S*) position in the azepane ring, also responds diversely to each ATP site which is reflected by its shape, conformational flexibility, and docking position of its moieties. In the nPKC isozymes where **1c** binds with higher affinities, PKCδ and PKCε, the azepane ring tend to have limited flexibility. On the contrary, where **1c** binds weaker in PKCη and PKCθ, more conformational fluctuations occur on the azepane ring. The way of the azepane ring behaves affecting the interactions of the other moieties, particularly the benzophenone moiety, with the ATP site residues.

Similar to our previous finding [50], the invariant Lys residue in the ribose subsite has major contribution on the binding of **1c**. The behavior of the azepane ring may strengthen or weaken the contribution of the invariant Lys. Furthermore, it may generate interactions of the benzophenone moiety with the ATP site residues either energetically favorable or unfavorable. We found that only the binding of **1c** to PKCε produce interactions with the ATP residues without causing unfavorable binding energy contribution, while optimizing binding contribution from the invariant Lys.

Overall, stereocontrolled fluorination in **1c** can harmonically tune with dynamics feature of PKCε but not the other nPKC isozymes. As a result, **1c** only shows cooperative interaction with the ATP site residues of PKCε, conferring selectivity for this isozymes. Therefore, the future design of balanol-based inhibitor need to maintain characteristic interactions made by **1c** and PKCε, such as optimal binding with the invariant Lys. This dynamic feature finding has implications for the rational design of balanol-based inhibitors targeting PKCε for cancer therapy.

## Additional file

Additional File 1: This file contains Notes 1 and 2, **Figures S1-S8.** and **Tables S1-S9.** and relevant references. (PDF 3690 kb)

## Abbreviations

AM1-BCC: Austin Model 1 - Bond Charge Corrections
ATP: Adenosine triphosphate;
Balanol: (−)-balanol
DOPE: Discrete Optimized Protein Energy
GAFF: General Amber Force-fields
LCPO: Linear Combination of Pairwise Overlaps
MD: Molecular Dynamics
MMGBSA: Molecular Mechanics Generalized Born Surface Area
nPKC: Novel Protein Kinase C
PCA: Principal Component Analysis
PKA: Protein Kinase A or cAMP-Dependent Protein Kinase
PKC: Protein Kinase C
PMEMD: Particle-Mesh Ewald Molecular Dynamics
RMSF: Root-Mean-Square Fluctuation
SASA: Solvent-Accessible Surface Area

## References

[1] Taylor SS, Yang J, Wu J, Haste NM, Radzio-Andzelm E, Anand G (2004). PKA: a portrait of protein kinase dynamics. Biochim Biophys Acta.

[2] Kulanthaivel P, Hallock YF, Boros C, Hamilton SM, Janzen WP, Ballas LM, Loomis CR, Jiang JB, Katz B (1993). Balanol: a novel and potent inhibitor of protein kinase C from the fungus Verticillium balanoides. J Am Chem Soc.

[3] Patel AR, Hardianto A, Ranganathan S, Liu F (2017). Divergent response of homologous ATP sites to stereospecific ligand fluorination for selectivity enhancement. Org Biomol Chem.

[4] Koide K, Bunnage ME, Gomez Paloma L, Kanter JR, Taylor SS, Brunton LL, Nicolaou KC (1995). Molecular design and biological activity of potent and selective protein kinase inhibitors related to balanol. Chem Biol.

[5] Cho YS, Lee YN, Cho-Chung YS (2000). Biochemical characterization of extracellular cAMP-dependent protein kinase as a tumor marker. Biochem Biophys Res Commun.

[6] Garg R, Benedetti LG, Abera MB, Wang H, Abba M, Kazanietz MG (2014). Protein kinase C and cancer: what we know and what we do not. Oncogene.

[7] Mochly-Rosen D, Das K, Grimes KV (2012). Protein kinase C, an elusive therapeutic target?. Nat Rev Drug Discov.

[8] Hu H, Mendoza JS, Lowden CT, Ballas LM, Janzen WP (1997). Synthesis and protein kinase C inhibitory activities of balanol analogues with modification of 4-hydroxybenzamido moiety. Bioorg Med Chem.

[9] Crane HM, Menaldino DS, Erik Jagdmann G, Darges JW, Buben JA (1995). Increasing the cellular PKC inhibitory activity of balanol: a study of ester analogs. Bioorg Med Chem Lett.

[10] Heerding JM, Lampe JW, Darges JW, Stamper ML (1995). Protein kinase C inhibitory activities of balanol analogs bearing carboxylic acid replacements. Bioorg Med Chem Lett.

[11] Lampe JW, Biggers CK, Defauw JM, Foglesong RJ, Hall SE, Heerding JM, Hollinshead SP, Hu H, Hughes PF, Jagdmann GE (2002). Synthesis and protein kinase inhibitory activity of Balanol analogues with modified benzophenone subunits. J Med Chem.

[12] Nicolaou KC, Koide K, Bunnage ME (1995). Total synthesis of Balanol and designed analogues. Chem Eur J.

[13] Gillis EP, Eastman KJ, Hill MD, Donnelly DJ, Meanwell NA (2015). Applications of fluorine in medicinal chemistry. J Med Chem.

[14] Hu X-G, Hunter L (2013). Stereoselectively fluorinated N-heterocycles: a brief survey. Beilstein J Org Chem.

[15] Hunter L (2010). The C-F bond as a conformational tool in organic and biological chemistry. Beilstein J Org Chem.

[16] Mortier J, Rakers C, Bermudez M, Murgueitio MS, Riniker S, Wolber G (2015). The impact of molecular dynamics on drug design: applications for the characterization of ligand-macromolecule complexes. Drug Discov Today.

[17] Hospital A, Goni JR, Orozco M, Gelpi JL (2015). Molecular dynamics simulations: advances and applications. Adv Appl Bioinforma Chem.

[18] Hardianto A, Liu F, Ranganathan S (2018). Molecular dynamics pinpoint the global fluorine effect in Balanoid binding to PKCε and PKA. J Chem Inf Model.

[19] UniProt C (2015). UniProt: a hub for protein information. Nucleic Acids Res.

[20] Roberts E, Eargle J, Wright D, Luthey-Schulten Z (2006). MultiSeq: unifying sequence and structure data for evolutionary analysis. BMC Bioinformatics.

[21] Humphrey W, Dalke A, Schulten K (1996). VMD: visual molecular dynamics. J Mol Graph.

[22] McWilliam H, Li W, Uludag M, Squizzato S, Park YM, Buso N, Cowley AP, Lopez R (2013). Analysis tool web services from the EMBL-EBI. Nucleic Acids Res.

[23] Taylor SS, Kornev AP (2011). Protein kinases: evolution of dynamic regulatory proteins. Trends Biochem Sci.

[24] Campanella JJ, Bitincka L, Smalley J (2003). MatGAT: an application that generates similarity/identity matrices using protein or DNA sequences. BMC Bioinformatics.

[25] Eswar Narayanan, Eramian David, Webb Ben, Shen Min-Yi, Sali Andrej (2008). Protein Structure Modeling with MODELLER. Methods in Molecular Biology.

[26] Shen MY, Sali A (2006). Statistical potential for assessment and prediction of protein structures. Protein Sci.

[27] Laskowski RA, MacArthur MW, Thornton JM: PROCHECK: Validation of protein structure coordinates. In: International Tables of Crystallography, Volume F Crystallography of Biological Macromolecules*.* Edited by Rossmann MG, Arnold ED, vol. F: Kluwer Academic Publishers; 2001: 722–725.

[28] Richter S, Wenzel A, Stein M, Gabdoulline RR, Wade RC (2008). webPIPSA: a web server for the comparison of protein interaction properties. Nucleic Acids Res.

[29] Madura JD, Briggs JM, Wade RC, Davis ME, Luty BA, Ilin A, Antosiewicz J, Gilson MK, Bagheri B, Scott LR (1995). Electrostatics and diffusion of molecules in solution: simulations with the University of Houston Brownian Dynamics program. Comput Phys Commun.

[30] Team RC (2015). R: a language and environment for statistical computing. R Foundation for Statistical Computing Vienna*,* Austria.

[31] Systèmes D (2016). Discovery studio visualizer. In*.*, v16.1.0.15350 edn.

[32] Jakalian A, Bush BL, Jack DB, Bayly CI (2000). Fast, efficient generation of high-quality atomic charges. AM1-BCC model: II. Parameterization and validation. J Comput Chem.

[33] Case DA, Betz RM, Botello-smith W, Cerutti DS, Cheatham TE, Darden TA, Duke RE, Giese TJ, Gohlke H, Goetz AW (2016). Amber 2016.

[34] Wang JM, Wolf RM, Caldwell JW, Kollman PA, Case DA (2004). Development and testing of a general Amber force field. J Comput Chem.

[35] Maier JA, Martinez C, Kasavajhala K, Wickstrom L, Hauser KE, Simmerling C (2015). ff14SB: improving the accuracy of protein side chain and backbone parameters from ff99SB. J Chem Theory Comput.

[36] Homeyer N, Horn AH, Lanig H, Sticht H (2006). AMBER force-field parameters for phosphorylated amino acids in different protonation states: phosphoserine, phosphothreonine, phosphotyrosine, and phosphohistidine. J Chem Theory Comput.

[37] Darden T, York D, Pedersen L (1993). Particle mesh Ewald: an N·log(N) method for Ewald sums in large systems. J Chem Phys.

[38] Fisette O, Wingbermuhle S, Tampe R, Schafer LV (2016). Molecular mechanism of peptide editing in the tapasin-MHC I complex. Sci Rep.

[39] Kumar A, Cocco E, Atzori L, Marrosu MG, Pieroni E (2013). Structural and dynamical insights on HLA-DR2 complexes that confer susceptibility to multiple sclerosis in Sardinia: a molecular dynamics simulation study. PLoS One.

[40] Ryckaert J-P, Ciccotti G, Berendsen HJC (1977). Numerical integration of the cartesian equations of motion of a system with constraints: molecular dynamics of n-alkanes. J Comput Phys.

[41] Berendsen HJC, Postma JPM, van Gunsteren WF, DiNola A, Haak JR (1984). Molecular dynamics with coupling to an external bath. J Chem Phys.

[42] Larini L, Mannella R, Leporini D (2007). Langevin stabilization of molecular-dynamics simulations of polymers by means of quasisymplectic algorithms. J Chem Phys.

[43] Pronk S, Páll S, Schulz R, Larsson P, Bjelkmar P, Apostolov R, Shirts MR, Smith JC, Kasson PM, van der Spoel D (2013). GROMACS 4.5: a highthroughput and highly parallel open source molecular simulation toolkit. Bioinformatics.

[44] Gohlke H, Case DA, Biology M, Scripps T (2004). Rd NTP: converging free energy estimates: MM-PB(GB)SA studies on the protein–protein complex Ras–Raf. J Comput Chem.

[45] Miller BR, McGee TD, Swails JM, Homeyer N, Gohlke H, Roitberg AE (2012). MMPBSA.Py: an efficient program for end-state free energy calculations. J Chem Theory Comput.

[46] Genheden S, Ryde U (2015). The MM/PBSA and MM/GBSA methods to estimate ligand-binding affinities. Expert Opin Drug Discov.

[47] Weiser J, Shenkin PS, Still WC (1999). Approximate atomic surfaces from linear combinations of pairwise overlaps (LCPO). J Comput Chem.

[48] Poongavanam V, Olsen JM, Kongsted J (2014). Binding free energy based structural dynamics analysis of HIV-1 RT RNase H-inhibitor complexes. Integr Biol (Camb).

[49] Hardianto A, Yusuf M, Liu F, Ranganathan S (2017). Exploration of charge states of balanol analogues acting as ATP-competitive inhibitors in kinases. BMC Bioinformatics.

[50] Hardianto A, Liu F, Ranganathan S (2017). Molecular dynamics pinpoint the global fluorine effect in balanoids binding to PKCε and PKA. J Chem Inf Model.

[51] Bakan A, Dutta A, Mao W, Liu Y, Chennubhotla C, Lezon TR, Bahar I (2014). Evol and ProDy for bridging protein sequence evolution and structural dynamics. Bioinformatics.

[52] Systèmes D (2016). BIOVIA Draw 2016.

[53] Team R (2016). RStudio: integrated development for R. in*.*, 0.99.892 edn.

[54] Wickham H (2009). ggplot2: Elegant graphics for data analysis. In.

[55] GIMP: GNU Image Manipulation Program 2.8.14 GPLv3. In.: https://www.gimp.org/; 2014.

[56] Inkscape: Inkscape 0.48.5 GPLv2. In*.*: https://inkscape.org/en/; 2014.

