# Supplementary material for: Diverse dynamics features of novel protein kinase C (PKC) isozymes determine the selectivity of a fluorinated balanol analogue for PKCε
Source: BMC Bioinformatics. 2019 Feb 4;19(Suppl 13):342. doi: 10.1186/s12859-018-2373-1 (PMC7394325; doi:10.1186/s12859-018-2373-1)
Supplement: Supplementary file 1 — This file contains Notes 1 and 2, Figures S1-S8. and Tables S1-S9. and relevant references. (PDF 3690 kb) [file 12859_2018_2373_MOESM1_ESM.pdf]

Additional File 1

Diverse dynamics features of novel protein kinase C (PKC) isozymes determine selectivity of a fluorinated balanol analogue for PKCε

Ari Hardianto, Varun Khanna, Fei Liu, Shoba Ranganathan

(*Email: shoba.ranganathan@mq.edu.au)

Note 1:	Charge state validation of **1c** in novel PKC isozymes................................2
Note 2:	Analysis of per-residue binding energy decomposition at the ATP sites....4
Figure S1.	(A) Trajectory of $\Delta G^{\circ}_{MMGBSA}$ of PKA- and nPKC isozymes-bound **1c** using chare state combination I and (B) correlation coefficients to experimental binding energy. 3
Figure S2.	(A) Trajectory of $\Delta G^{\circ}_{MMGBSA}$ of PKA- and nPKC isozymes-bound **1c** using chare state combination II and (B) correlation coefficients to experimental binding energy. 3
Figure S3.	(A) Sequence alignment of PKA and novel PKC isozymes on their ATP sites and (B) heat map of their clustering based on Molecular Surface Electrostatic Potential (MSEP)...8
Figure S4.	Root mean square fluctuation (RMSF) of the *apo* and bound forms of nPKC isozymes. (A) and (B) are overlaid RMSF plots of nPKC isozymes in the *apo* and bound forms, respectively...9
Figure S5.	Structural mapping of principal component analysis (PCA) dynamics modelling on the *apo* and bound kinases...9
Figure S6.	Polar charts of dihedral angles on the azepane ring. 10
Figure S7.	Per-residue binding energy contributions of the ATP site of novel PKC isozymes to 1c binding. 11
Figure S8.	Interactions of 1c with residues in the ATP sites of nPKC isozymes. 12
Table S1.	PKC isozymes and PKA..13
Table S2.	Cosine contents of eight principal components calculated from MD trajectories of both the *apo* and bound forms of nPKC isoforms...............14
Table S3.	Sequence similarity and identity matrix of the ATP site residues of novel PKC isozymes..15
Table S4.	Sequence similarity and identity of kinase domains of novel PKC isozymes. 15
Table S5	Comparison between RMSF values of kinases in the *apo* and bound forms using a Wilcoxon sum-ranked test. 16
Table S6.	RMSF changes of kinases after ligand binding to their ATP sites. 16
Table S7.	SASA of novel PKC isozymes and PKA in the *apo* and bound forms. 17
Table S8.	H-bond conservation..18
Table S9.	$\Delta G^{\circ}_{MMGBSA}$ of natural balanol 1 and its C5(S)-fluorinated analogue (**1c**) to PKC isozymes and PKA...19

Note 1: Charge state validation of 1c in novel PKC isozymes

Our previous investigation proposed that **1c** has same charge state when it is bound to the ATP site of PKCε or PKA [1]. Balanoid **1c** has a positive formal charge on the amine (N1) group on the azepane ring. Additionally, negative formal charges present on the phenol (C6″OH) and carboxylic (C15″OOH) groups of the benzophenone moiety. Thus, we assigned these charges to PKCδ-, PKCε-, PKCη-, and PKCθ-bound **1c** (annotated as charge combination I). To increase the number of data points for charge state validation, we also included PKA-bound **1c** to charge combination I. Each kinase-**1c** complex was then subjected to molecular dynamics simulation. Binding energy value of each complex was calculated using MMGBSA approach within 10-ns-sliding-window every 10 ns. The resulting $\Delta G^{\circ}_{MMGBSA}$ values were evaluated further by plotting them over the MD trajectory and calculating their correlation coefficients with experiment.

The $\Delta G^{\circ}_{MMGBSA}$ profile (Figure S1.A) shows that binding energy values of PKCδ-bound **1c** are outliers, even though the r^2 plot depicts good correlations, above 0.70 from 40 to 90 ns (Figure S1.B). The experimental binding affinity order for **1c** to kinase is PKCε > PKCδ ≈ PKA > PKCη ≈ PKCθ. The $\Delta G^{\circ}_{MMGBSA}$ values of **1c** bound to PKCδ, however, are close to those of PKCη- and PKCθ-bound **1c** (Figure S1.A). We then revisited the charge state of PKCδ-bound **1c**. Our preceding study on charge state exploration of fluorinated balanoids suggests that local environment of the ATP site may influence the charge state of the phenol (C6″OH) group, where it can be charged or neutral [1]. According to this result, we assigned C6″OH neutral for PKCδ-bound **1c**, whereas charge states of the remaining isozyme-bound **1c** molecules are unchanged. We labeled those charge states as charge state combination II and run another MD simulation. The resulting $\Delta G^{\circ}_{MMGBSA}$ profile shows the calculated binding energy values of PKCδ-bound **1c** moves closer to those of PKA-bound **1c** (Figure S2.A), which is expected. Moreover, after 30 ns of trajectory, correlation coefficients are above 0.80 (Figure S2.B), with

average of 0.90. Thus, in further analysis, we used **1c** bearing charges on N1, C6″OH, and C15″OOH in kinases studied, except in PKCδ. In PKCδ, **1c** has charges on N1 and C15″OOH.

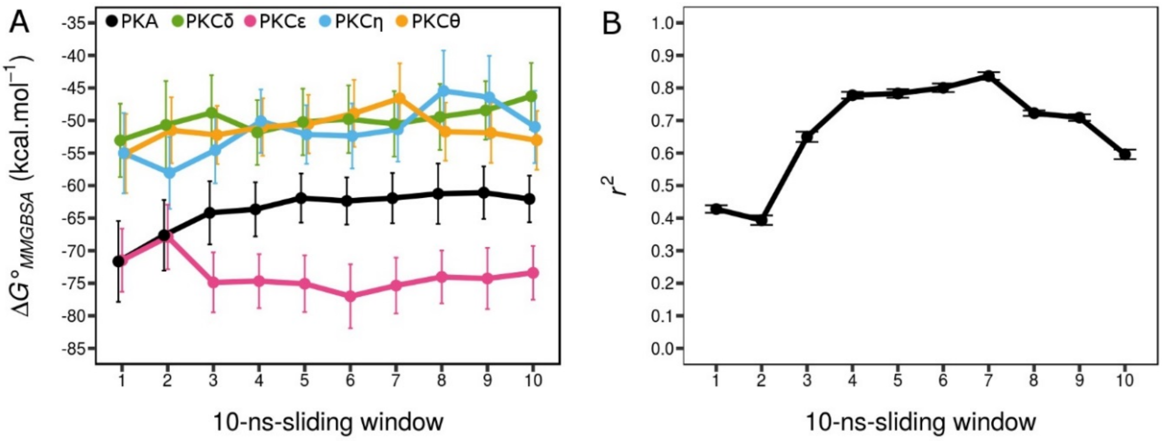

Figure S1. (A) Trajectory of $\Delta G^{\circ}_{MMGBSA}$ of PKA- and nPKC isozymes-bound 1c using chare state combination I and (B) correlation coefficients to experimental binding energy.

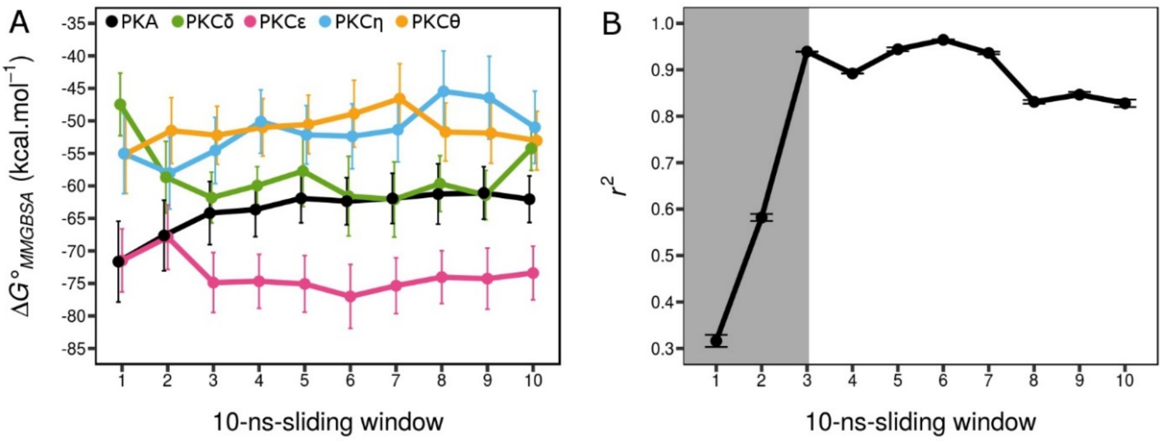

Figure S2. (A) Trajectory of $\Delta G^{\circ}_{MMGBSA}$ of PKA- and nPKC isozymes-bound 1c using chare state combination II and (B) correlation coefficients to experimental binding energy.

Note 2: Analysis of per-residue binding energy decomposition at the ATP sites

General overview

Decomposition analysis of per-residue binding energy at the ATP site (Figure S7) suggests that novel PKC isozymes contribute differently to **1c**. The only exception is the contribution from the adenine subsite where the benzamide moiety shows similar docking position (main paper: Figure 2). The adenine subsite residues also show similar H-bond profiles with the benzamide moiety (Table S8), particularly among novel PKC isozymes.

In the ribose subsite, two residues among kinases studied exhibit different binding energy contributions to **1c** binding (Figure S7). The most dispersed contributions are ranging from -3.24 to -18.01 kcal.mol^{-1}, where the strongest binding comes from Asp491 in PKCδ and the weakest from the corresponding residue Asp522 in PKCθ. The second residue contributes varied binding energy values within a range -2.17 (Asp483 in PKCη) to -6.06 kcal.mol^{-1} (Asp477 in PKCδ). The binding energy strength may correlate to H-bond conservation with the amine N1 group of the azepane ring in **1c**. For example, Asp491 in PKCδ has a fully conserved H-bond (99.3%), whereas Asp522 does not forms the H-bond with the amine group (Table S8).

As the regions where the flexible Gly-rich loop is mainly located, the residues of the triphosphate subsite also show diverse binding energy contributions to **1c** binding among the given kinases. In particular, the invariant Lys provides the most varied contributions (-6.34 to -16.83 kcal.mol^{-1}). Additionally, this residue frequently provides the strongest binding energy contribution among the other residues in the ATP sites of the kinases, except in the ATP site of PKCδ. The other corresponding residues for Glu403 in PKCη and Glu428 in PKCθ, which cause repulsive binding to **1c** (Figure S7), also shows diverse binding energy values (-2.66 ± 3.60 kcal.mol^{-1}).

As mentioned in the paragraph above, the invariant Lys residues among novel PKC isozymes provides the strongest binding energy contribution to **1c**. This result is similar to our previous study [2] that the invariant Lys residue in PKA and PKCε contribute a major binding contribution to the binding of balanoids studied. The invariant Lys can provide four different ways to non-covalently bind **1c**, which include H-bond, charge-charge, alkyl-π hydrophobic, and cation-π interactions. The following discussions are presenting the effect of the dynamics features of nPKC isozymes on the interactions between the invariant Lys and **1c** as well as binding energy contributed by this residue.

The invariant Lys residue contributions

The phenolate C6″O⁻ group of **1c** binds to the invariant Lys437 in PKCε through four ways: alkyl-π hydrophobic, cation-π, a H-bond, and charge-charge interactions. Additionally, Lys437 the and carbonyl C1'=O of **1c** forms a H-bond with moderate conservation (44.0%; Table S8). As a consequence, **1c** acquires a very strong binding contribution of -21.39 kcal.mol^{-1} from Lys437 (Figure S7).

In PKCη and PKCθ, each of which has different dynamics behaviour to PKCε, balanoid **1c** obtains weaker binding energy contributions from the invariant Lys residues. The invariant Lys in both kinases builds interactions of alkyl-π hydrophobic, cation-π, and transient H-bond with the phenolate C6″O⁻ group in **1c**. However, the H-bond between C1'=O and the invariant Lys residues (Lys363 in PKCη and Lys409 in PKCθ) is absent. Such interactions made by **1c**, in the ATP site of PKCη and PKCθ, cause Lys363 and Lys409 respectively, to consecutively provide weaker binding contributions of -6.34 and -8.38 kcal.mol^{-1} (Figure S7). The greater (i.e. more negative) binding energy contribution by Lys409 is due to the H-bond between the invariant Lys and C10"-OH with conservation of 16.2% (Table S8).

The invariant Lys378 in PKCδ provides a relatively weaker binding contribution to **1c**. This may be because the unique dynamics of PKCδ and the uncharged phenolic C6″OH group which

leads to fewer possibilities for interacting with Lys378. The group non-covalently binds Lys378 only through an alkyl-π hydrophobic interaction and very a transient H-bond (0.4% conservation, Table S8). As a result, **1c** only obtains -8.79 kcal.mol^{-1} from Lys378 when bound to PKCδ. However, in the ribose subsite, Asp491 stabilizes the binding of **1c** with -18.01 kcal.mol^{-1}. This binding energy contribution is associated with a conserved H-bond (99.3%) and a salt bridge between the negatively charged side chain of Asp491 and the protonated N1 amine group in the azepane ring (main paper: Figure 5.B).

Unfavorable binding energy

Diverse dynamics of nPKC isozymes affect **1c** binding in terms of shape, conformational bias, docking mode (main paper: Figure 2), and also interactions with the residues in the ATP sites. These different effects may implicate in unfavorable binding energy contributions that weaken the affinity of 1c to novel PKC isozymes. For example, Glu403 in PKCη contributes a repulsive binding energy of 0.14 kcal.mol^{-1} to **1c**, whereas Asp421 and Glu428 in PKCθ serves 0.01 and 0.06 kcal.mol^{-1} (Figure S7), respectively. In PKCδ, the azepane ring of **1c** involves in a repulsive binding energy (0.19 kcal.mol^{-1}) with Lys475 in the ribose subsite. This unfavorable binding energy violates the strong binding energy contributed (-18.01 kcal.mol^{-1}) by Asp491 (Figure S7), reducing the binding affinity of **1c** to PKCδ.

Overall decomposition analysis of per-residue binding energy suggests that although the ATP site residues among novel PKC isozymes are highly similar, the different dynamics of novel PKC isozymes result in dispersed profiles of binding energy contributions (Figure S7) to **1c**. Interestingly, while the binding of **1c** to PKCδ, PKCη, and PKCθ cause unfavourable binding interactions, PKCε-bound **1c** is free from such binding interaction. Additionally, the binding of **1c** to PKCε optimize the interactions with invariant Lys437 which was also reported in our previous work [2]. Hence, C5(*S*)-fluorine perturbation in **1c** sets up properly the azepane ring and remotely drives the conformation of the benzophenone moiety to optimally interact

with residues in the triphosphate subsite, particularly invariant Lys, which is only possible with PKCε. As a result, balanoid **1c** has a high affinity and is selective to PKCε over PKA, other novel PKC isozymes, and also classical as well as atypical PKC isozymes (as suggested by $\Delta G^{\circ}_{MMGBSA}$ calculation in Table S8).

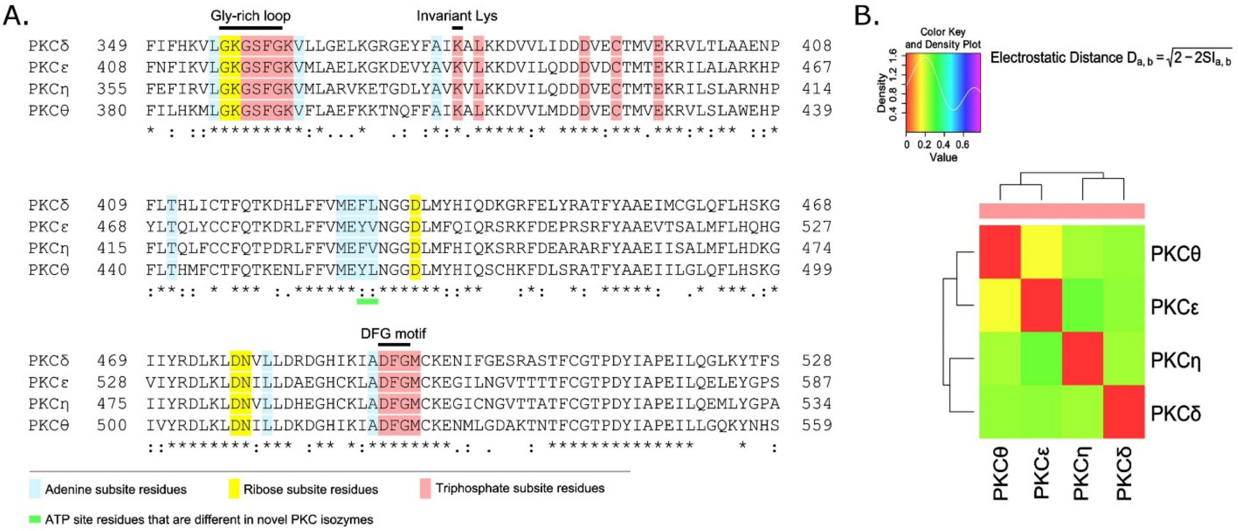

Figure S3. (A) Sequence alignment of PKA and novel PKC isozymes on their ATP sites and (B) heat map of their clustering based on Molecular Surface Electrostatic Potential (MSEP). Blue, yellow, and red-shaded colors in (A) highlight adenine subsite, ribose, and triphosphate residues, respectively. Green bars under alignment indicate the ATP site residues of PKA that are different to those of novel PKC isozymes. ***** - identical residue; **:** - conservative substitution; **.** – non-conservatively substituted.

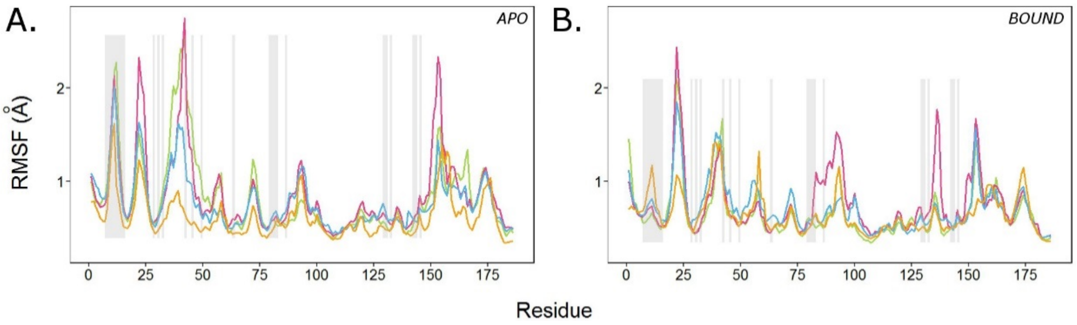

Figure S4. Root mean square fluctuation (RMSF) of the *apo* and bound forms of nPKC isozymes. (A) and (B) are overlaid RMSF plots of nPKC isozymes in the *apo* and bound forms, respectively. Residues of ATP binding sites are indicated by grey-shaded bars. Residue numbering restarts from zero for all nPKC isoforms. PKCδ, ε, η, and θ are represented by green, magenta, blue, and orange color, respectively.

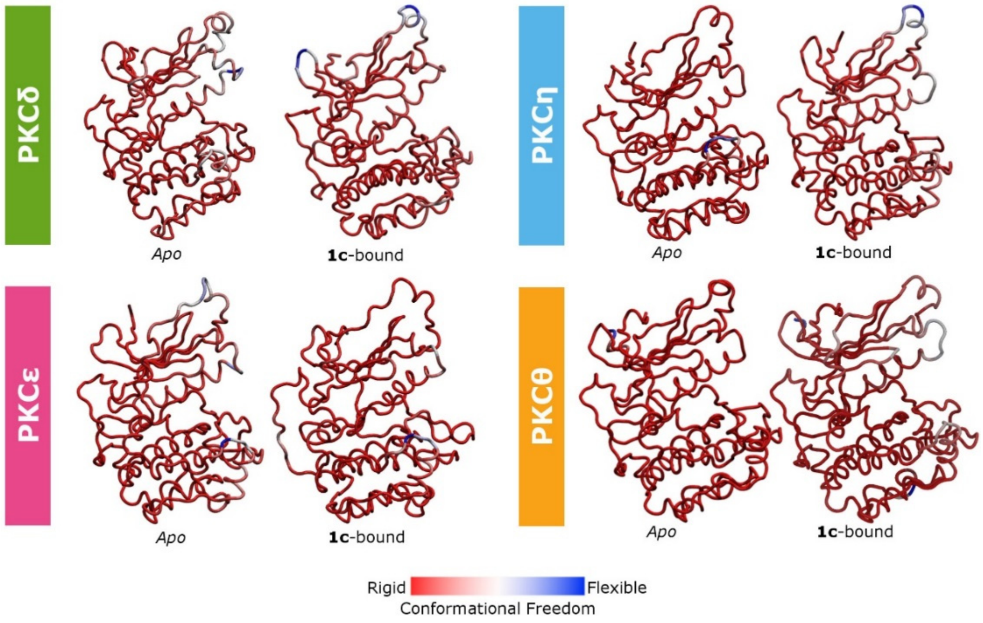

Figure S5. Structural mapping of principal component analysis (PCA) dynamics modelling on the *apo* and bound kinases. Principal modes of MD trajectories are calculated using singular value decomposition (SVD). Coloring scale of protein conformational freedom runs from red to blue which represents rigid to high flexibility.

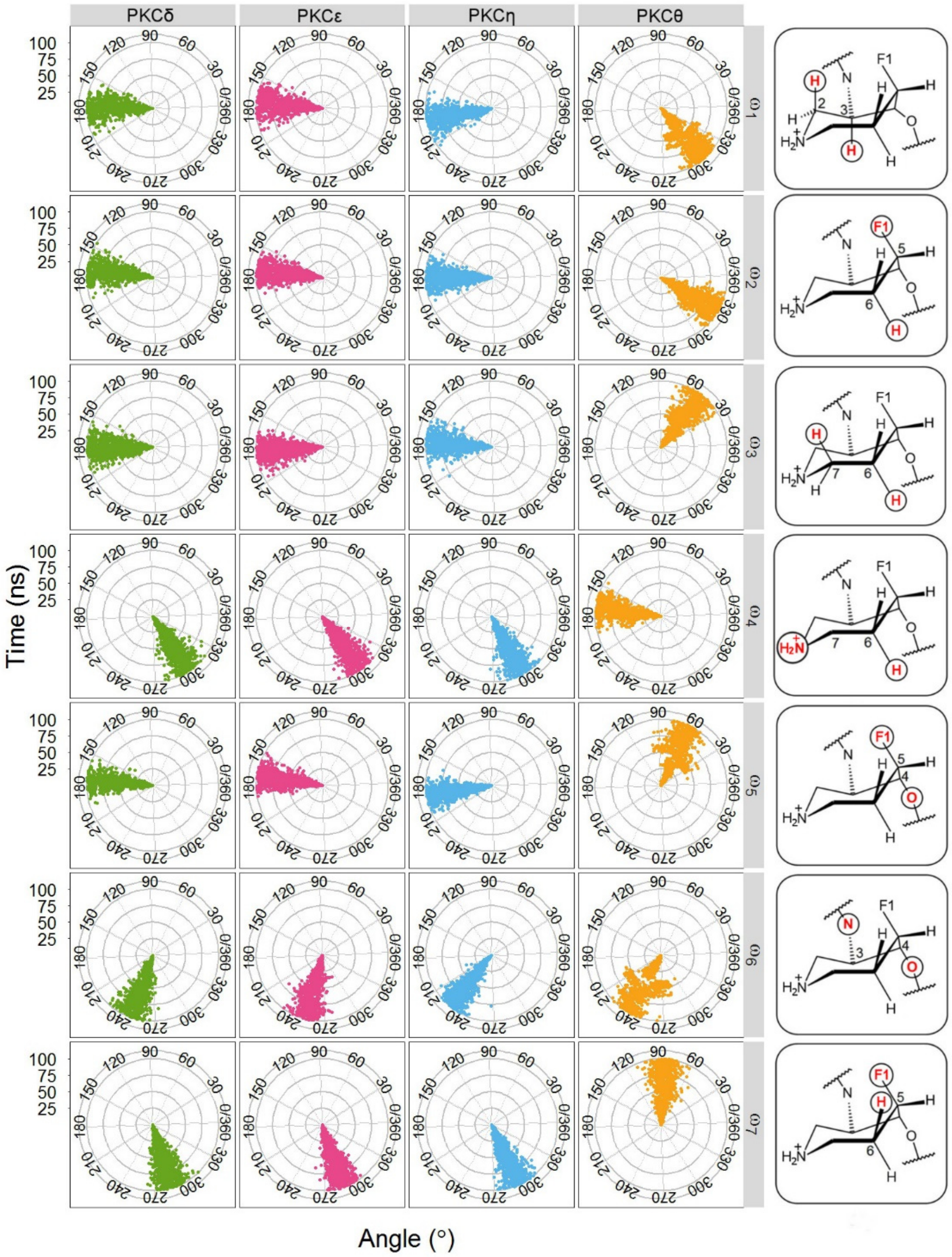

Figure S6. Polar charts of dihedral angles on the azepane ring. Dihedral angle plots on each column represent the azepane ring of kinase-bound **1c,** for the last 100 ns of MD simulation. Plots on rows monitor dihedral angles on the azepane ring that are indicated by red color atoms in the structures on the right panel.

Moiety	C5(*S*)-Fluorinated analogue 1c				C5(*S*)-Fluorinated analogue 1c					
	Residue				ΔG° Contribution (kcal.mol^{-1})					
	PKCδ	PKCε	PKCη	PKCθ	PKCδ	PKCε	PKCη	PKCθ	Mean	SD
Benzophenone	Gly358	Gly417	Gly364	Gly389	-5.59	-4.25	-5.08	-5.45	-5.09	0.60
	Ser359	Ser418	Ser365	Ser390	-8.19	-4.12	-8.00	-7.69	-7.00	1.93
	Phe360	Phe419	Phe366	Phe391	-5.17	-5.58	-4.55	-5.91	-5.30	0.59
	Gly361	Gly420	Gly367	Gly392	-2.11	-2.59	-1.44	-2.28	-2.11	0.49
	Lys362	Lys421	Lys368	Lys393	-1.98	-2.64	-1.89	-1.71	-2.06	0.41
	**Lys378**	**Lys437**	**Lys384**	**Lys409**	-8.79	-16.83	-6.34	-8.38	-10.09	4.62
	Leu380	Leu439	Leu386	Leu411	-2.62	-3.17	-2.64	-2.80	-2.81	0.25
	Asp390	Asp449	Asp396	Asp421	-0.04	-0.20	-1.47	0.01	-0.43	0.70
	Cys393	Cys452	Cys399	Cys424	-1.28	-1.41	-0.51	-1.15	-1.09	0.40
	Glu397	Glu456	Glu403	Glu428	-3.37	-7.46	0.14	0.06	-2.66	3.60
	Gly493	Gly552	Gly499	Gly524	-1.09	-1.34	-1.46	-1.13	-1.26	0.18
Azepane	Gly356	Gly415	Gly362	Gly387	-1.68	-2.73	-2.43	-3.47	-2.58	0.74
	Lys357	Lys416	Lys363	Lys388	-3.87	-3.89	-2.47	-2.95	-3.30	0.70
	Asp434	Asp493	Asp440	Asp465	-0.92	-0.09	-0.18	-0.65	-0.46	0.39
	Lys475	Lys534	Lys481	Lys506	0.19	-0.17	-0.84	-0.36	-0.30	0.43
	Asp477	Asp536	Asp483	Asp508	-6.06	-2.66	-2.17	-3.01	-3.48	1.76
	Asn478	Asn537	Asn484	Asn509	-0.76	-1.99	-2.87	-1.43	-1.76	0.89
	Asp491	Asp550	Asp497	Asp522	-18.01	-5.88	-3.77	-3.24	-7.73	6.95
Benzamide	Leu355	Leu414	Leu361	Leu386	-1.55	-1.83	-1.86	-2.40	-1.91	0.36
	Val363	Val422	Val369	Val394	-4.07	-4.76	-4.88	-4.08	-4.45	0.43
	Ala376	Ala435	Ala382	Ala407	-1.30	-1.42	-1.50	-1.36	-1.40	0.09
	Thr411	Thr470	Thr417	Thr442	-0.57	-0.80	-0.87	-0.84	-0.77	0.14
	Met427	Met486	Met433	Met458	-2.07	-1.69	-1.72	-1.70	-1.80	0.18
	Glu428	Glu487	Glu434	Glu459	-2.87	-2.75	-3.12	-3.40	-3.04	0.29
	Phe429	Tyr488	Phe435	Tyr460	-0.70	-1.94	-1.58	-1.70	-1.48	0.54
	Leu430	Val489	Val436	Leu461	-2.37	-2.33	-1.77	-2.22	-2.17	0.28
	Leu480	Leu539	Leu486	Leu511	-2.55	-2.57	-2.57	-3.12	-2.70	0.28
	Ala490	Ala549	Ala496	Ala521	-0.95	-1.94	-2.15	-2.08	-1.78	0.56
	ΔG° MMGBSA (kcal.mol^{-1})				-45.84	-70.35	-42.35	-39.77		
	K_d (nM)				4.9	0.4	13	24		

-22 -21 -20 -19 -18 -17 -16 -15 -14 -13 -12 -11 -10 -9 -8 -7 -6 -5 -4 -3 -2 -1 0 1 kcal.mol^{-1}

Figure S7. Per-residue binding energy contributions of the ATP site of novel PKC isozymes to 1c binding.

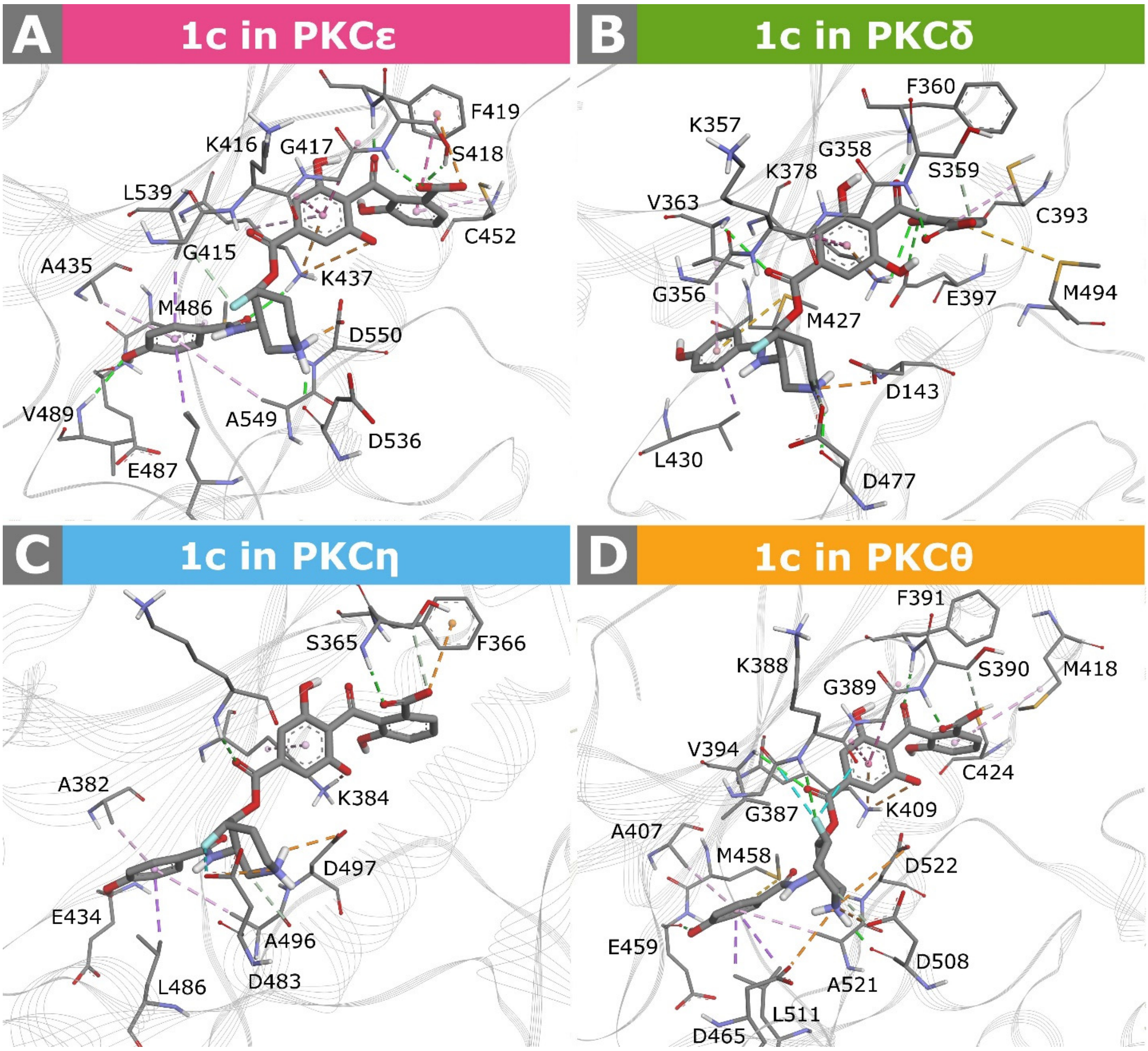

Figure S8. Interactions of 1c with residues in the ATP sites of nPKC isozymes.

Table S1. PKC isozymes and PKA. The table contains information of Uniprot ID, template(s) used in homology modelling, and phosphorylation sites required for full activation of PKC isozymes [3].

Isozyme	Uniprot ID	Template(s)*	Phosphorylation sites		
			Activation loop	Turn motif	Hydrophobic motif
PKCα	P17252	3IW4, 4RA4, 1BX6	Thr497	Thr638	Ser657
PKCβI	P05771	2I0E, 1BX6	Thr500	Thr642	Ser661
PKCβII	P05771-2	2I0E, 1BX6	Thr500	Thr642	Ser660
PKCγ	P05129	2I0E, 1BX6	Thr514	Thr655	Ser674
PKCδ	Q05655	1BX6, 5F9E	Thr507	Ser645	Ser664
PKCε	Q02156	1BX6, 3TXO	Thr566	Thr710	Ser629
PKCη	P24723	3TXO, 1BX6, 4RA4	Thr513	Thr656	Ser675
PKCθ	Q04759	1BX6, 5F9E	Thr538	Ser676	Ser695
PKCι	P41743	1BX6, 5LI9, 4DC2	Thr412	Thr564	-
PKCζ	Q04759	1BX6, 5LI9, 4DC2	Thr410	Thr560	-
PKA	P17612	1BX6	Thr198	Ser339	-

* The codes listed in Template(s) column are Protein Data Bank (PDB) ID

Homology modelling and MD simulations for PKCα, βI, βII, γ, ι, and ζ were conducted as described in sub-sections of **Homology modelling** and **Molecular dynamics simulation preparation and protocol** in **Materials and Methods**. Phosphoryl moiety was added to each kinase as listed in Table S1.

Table S2. Cosine contents of eight principal components calculated from MD trajectories of both the *apo* and bound forms of nPKC isoforms.

	Apo				Bound			
	**PKCδ**	**PKCε**	**PKCη**	**PKCθ**	**PKCδ**	**PKCε**	**PKCη**	**PKCθ**
Trajectory range (ns)	100-300	100-300	150-300	175-300	175-300	330-470	100-300	200-300
PC1	0.054	0.212	0.175	0.375	0.368	0.024	0.194	0.385
PC2	0.008	0.100	0.099	0.225	0.017	0.013	0.000	0.162
PC3	0.030	0.079	0.000	0.000	0.000	0.101	0.042	0.267
PC4	0.044	0.071	0.035	0.061	0.030	0.038	0.021	0.106
PC5	0.083	0.265	0.003	0.078	0.007	0.002	0.000	0.104
PC6	0.004	0.124	0.048	0.017	0.001	0.028	0.017	0.004
PC7	0.014	0.058	0.015	0.031	0.017	0.033	0.021	0.181
PC8	0.008	0.008	0.049	0.063	0.013	0.000	0.000	0.002

In order to obtain meaningful analyses, we monitored MD trajectories using cosine contents of eight principal components. Cosine content values range from 0 to 1, where values close to 1 is the sign of nonconverged trajectories due to random diffusion [4]. Low cosine content values may indicate converged trajectories. The majority of our MD simulations reach low cosine content after 100 ns. Based on cosine content values, we generated 300 ns trajectories for all MD simulations, except for the bound form of PKCε (470 ns).

Table S3. Sequence similarity and identity matrix of the ATP site residues of novel PKC isozymes. Matrix Global Alignment Tool (MatGAT) [5] was used to generated the matrix.

		Identity (%)			
		PKCδ	PKCε	PKCη	PKCθ
Similarity (%)	PKCδ		92.9	96.4	96.4
	PKCε	100		96.4	96.4
	PKCη	100	100		92.9
	PKCθ	100	100	100	

Table S4. Sequence similarity and identity of kinase domains of novel PKC isozymes. Matrix Global Alignment Tool (MatGAT) [5] was used to generated the matrix.

		Identity (%)			
		PKCδ	PKCε	PKCη	PKCθ
Similarity (%)	PKCδ		61.9	61.7	76.3
	PKCε	78.5		76.7	60.4
	PKCη	76.9	89.3		59.5
	PKCθ	90.2	78.9	77.3	

Table S5 Comparison between RMSF values of kinases in the *apo* and bound forms using a Wilcoxon sum-ranked test.

Kinase	*p*-Value		
	Kinase domain	Gly-rich loop	ATP site
PKCδ	4.21×10^{-4}	2.16×10^{-3}	9.73×10^{-2}
PKCε	3.49×10^{-2}	2.16×10^{-3}	6.27×10^{-3}
PKCη	3.32×10^{-4}	2.16×10^{-3}	3.12×10^{-2}
PKCθ	2.03×10^{-2}	5.89×10^{-1}	2.00×10^{-1}

Table S6. RMSF changes of kinases after ligand binding to their ATP sites. Negative sign indicates RMSF reduction occurs after ligand binding, whereas a positive sign indicates an increase in RMSF after ligand binding.

Kinase	ΔRMSF (Å)					
	Kinase domain	Gly-rich loop	ATP site	triphosphate	ribose	adenine
PKCδ	-0.211	-1.067	-0.393	-0.839	-0.235	-0.058
PKCε	-0.063	-0.847	-0.310	-0.743	-0.095	-0.028
PKCη	-0.104	-0.205	-0.161	-0.403	-0.161	-0.038
PKCθ	0.017	-0.184	0.004	-0.036	0.000	0.048

Table S7. SASA of novel PKC isozymes and PKA in the *apo* and bound forms. The values listed are average SASA values, in Å^2.

State	Isozymes	Mean	SD	Median	Min.	Max.
Apo	PKCδ	2614.13	55.73	2603.72	2479.66	2813.56
	PKCε	2598.62	58.72	2602.56	2405.16	2751.28
	PKCη	2682.26	44.97	2683.86	2531.21	2822.90
	PKCθ	2636.96	43.13	2638.59	2463.41	2769.97
**1c-bound**	PKCδ	2485.84	47.88	2484.84	2348.08	2652.92
	PKCε	2276.31	38.26	2273.34	2163.74	2406.57
	PKCη	2406.86	44.46	2405.99	2280.22	2584.70
	PKCθ	2476.39	87.25	2452.59	2301.24	2742.77

Table S8. H-bond conservation. The table lists H-bond persistence of **1c** with the ATP site residues of nPKC isozymes.

**1c**			**PKCδ**		**PKCε**		**PKCη**		**PKCθ**	
**Group**	**M**[a]	**R**[b]	**Residue**	**%**	**Residue**	**%**	**Residue**	**%**	**Residue**	**%**
C6F1	Az	BB		-		-		-	Lys388	24.1
C15"OOH	Bp	BB	Ser359	64.0	Ser418	15.5	Ser365	71.1	Ser390	76.7
C15"OOH	Bp	SC		74.2		8.5		68.2		67.9
C8"=O	Bp	BB	Phe360	54.2	Phe419	19.9	Phe366	30.8	Phe391	28.3
C15"OOH	Bp	BB		-		15.0		9.2		17.1
C15"OOH	Bp	BB		-		-	Gly367	0.6	Gly392	5.4
C1'=O	Am	SC		-	Lys437	44.0		-		-
C8"=O	Bp	SC		-		-		-	Lys409	9.1
C6"-OH	Bp	SC	Lys378	0.4		0.5	Lys384	6.8		6.7
C10"-OH	Bp	SC		0.2		1.7		0.7		16.2
C10"-OH	Bp	SC	Glu397	50.0	Glu456	100.0	Glu403	10.3		-
C5'-OH	Bm	BB	Glu428	64.8	Glu487	89.3	Glu434	87.3	Glu459	88.1
C5'-OH	Bm	BB	Leu430	4.9	Val489	9.9	Val436	23.3	Leu461	10.6
N1	Az	BB	Asp477	46.4	Asp536	30.8	Asp483	0.3	Asp508	41.9
N1	Az	BB	Asn478	7.0		-	Asn484	0.8	Asn509	1.4
N1	Az	SC		7.3	Asn537	15.5		8.1		-
C6"-OH	Bp	SC	Asp491	6.4		-				-
N1	Az	SC		99.3	Asp550	66.0	Asp497	19.2		-
C5'-OH	Bm	SC	Tyr630	3.0		-		-		-
K_d (nM)			4.9 ± 0.40		0.4 ± 0.01		13.0 ± 0		24.0 ± 0.50	

[a]M is moiety including Bp or benzophenone, Bm benzamide, and Az azepane, whereas Am is amide linkage.

[b]R is the parts of residue interacting with ligand: BB is backbone and SC is side chain

Table S9. $\Delta G^{\circ}_{MMGBSA}$ of natural balanol 1 and its C5(S)-fluorinated analogue (1c) to PKC isozymes and PKA.

**Kinase**	$\Delta G^{\circ}_{MMGBSA}$ **(kcal.mol^{-1})**	
	**(-)-Balanol 1**	**1c**
PKA	-66.84	-63.77
PKCα	-67.64	-36.42
PKCβI	-62.44	-48.92
PKCβII	-56.76	-45.17
PKCγ	-73.21	-56.79
PKCδ	-72.92	-45.84
PKCε	-67.52	-70.35
PKCη	-63.34	-42.35
PKCθ	-67.51	-39.77
PKCι	-55.47	-44.28
PKCζ	-54.04	-39.34

$\Delta G^{\circ}_{MMGBSA}$ computations suggest that stereospecific fluorination on balanol at C5(*S*) results in binding selectivity to PKCε. As seen in Table S6, **1c** has decreased binding affinity to all PKC isozymes, except PKCε.

REFERENCES

1. Hardianto A, Yusuf M, Liu F, Ranganathan S: **Exploration of charge states of balanol analogues acting as ATP-competitive inhibitors in kinases**. *BMC Bioinformatics* 2017, **18**:Suppl 16.
2. Hardianto A, Liu F, Ranganathan S: **Molecular Dynamics Pinpoint the Global Fluorine Effect in Balanoid Binding to PKCε and PKA**. *Journal of Chemical Information and Modeling* 2018, **58**(2):511-519.
3. UniProt C: **UniProt: a hub for protein information**. *Nucleic Acids Research* 2015, **43**(Database issue):D204-212.
4. Hess B: **Similarities between principal components of protein dynamics and random diffusion**. *Physical Review E* 2000, **62**(6):8438-8448.
5. Campanella JJ, Bitincka L, Smalley J: **MatGAT: An application that generates similarity/identity matrices using protein or DNA sequences**. *BMC Bioinformatics* 2003, **4**(1):29.
